# Genetic variants of LncRNAs HOTTIP and MEG3 influence nasopharyngeal carcinoma susceptibility and clinicopathologic characteristics in the Southern Chinese population

**DOI:** 10.1186/s13027-024-00591-6

**Published:** 2024-07-24

**Authors:** Xiaoxia Lao, Yujie Wang, Rongxin Huang, Yanying He, Huabiao Lu, Dan Liang

**Affiliations:** 1 Department of Clinical Laboratory, Minzu Hospital of Guangxi Zhuang Autonomous Region, Affiliated Minzu Hospital of Guangxi Medical University, Guangxi, China; 2 Department of Otolaryngology, Minzu Hospital of Guangxi Zhuang Autonomous Region, Affiliated Minzu Hospital of Guangxi Medical University, Guangxi, China

**Keywords:** Nasopharyngeal carcinoma, lncRNA, HOTTIP, MEG3, Polymorphism, Genotypes

## Abstract

**Objective:**

Recent studies have indicated that HOTTIP and MEG3 are associated with the initiation and progression of various types of tumors, including nasopharyngeal carcinoma (NPC). This investigation aimed to elucidate the impact of HOTTIP and MEG3 polymorphisms on the susceptibility and clinicopathologic characteristics of NPC.

**Methods:**

This research employed next-generation sequencing and multiplex PCR to assess the polymorphisms of HOTTIP rs1859168 and MEG3 rs7158663 in 200 NPC and 200 healthy individuals respectively. HOTTIP and MEG3 expression were assessed *via* qRT-PCR assessment. Furthermore, the genotypes and alleles frequency of rs1859168 and rs7158663 were compared between healthy and NPC individuals to elucidate their influence on NPC susceptibility and relation with clinicopathologic characteristics.

**Results:**

In comparison with the healthy cohort, the presence of HOTTIP rs1859168 CC genotype and the C allele were markedly linked with increased NPC incidence (*p < 0.05*). Furthermore, the MEG3 rs7158663 AA genotype and the A allele also indicated an increased risk of NPC (*p < 0.05*). The subgroup analysis of age, EBV infection, gender, nationality, smoking, and drinking status revealed no marked association between rs1859168 and rs7158663 genotypes and these potential confounding factors. Moreover, it was observed that rs1859168 CC and rs7158663 AA genotypes were related to local tumor invasion and lymph node metastasis. Additionally, HOTTIP indicated a marked elevation, while MEG3 substantially reduced in NPC samples than the normal nasopharyngeal biospecimens. Patients who carried CC or CA genotypes rather than the HOTTIP rs1859168 AA genotype, had substantially higher HOTTIP levels, while patients with rs7158663 AA or GA genotypes indicated notably lower expression of MEG3 than GG genotype carriers.

**Conclusion:**

Individuals with genetic variants of HOTTIP rs1859168 and MEG3 rs7158663 might have an increased risk of NPC susceptibility and related clinicopathologic characteristics, potentially by affecting the expression of HOTTIP and MEG3.

## Introduction

Nasopharyngeal carcinoma (NPC) is a malignant tumor that is distributed in various geographical and ethnic regions and has an increased incidence rate in Southern China [1]. It has a complex etiology, however, infection by Epstein-Barr virus (EBV) is the primary cause. EBV transforms normal host cells into cancerous types and can stimulate or inhibit various host cellular processes and mechanisms. Furthermore, NPC is not only influenced by genetics, but also environmental risk factors, including prolonged cigarette smoking, drinking, and various dietary factors. However, different individuals with the same environmental risk factors present distinct characteristics, indicating that in NPC formation, genetic susceptibility is the primary risk factor. Single nucleotide polymorphism (SNP) is the most common type of heritable variation in humans, which can affect individual susceptibility to tumors and is essentially linked to tumor pathogenesis, advancement, and prognosis [2].

Long non-coding RNAs (lncRNAs) comprise more than 200 nucleotides and regulate various cellular processes at the post-transcriptional, epigenetic, and transcriptional levels. Recently, it has been indicated that abnormal lncRNA levels are linked with the initiation, metastasis, susceptibility, and prognosis of multiple human cancers, such as gastric cancer, breast cancer, and NPC [3–5]. According to GWAS, only 7% of disease-related loci are present in protein-coding regions, while 93% are in non-coding regions, indicating that genetic alterations in non-coding sites may play a crucial part in regulating disease progression [6]. Furthermore, the literature has revealed that SNPs in lncRNA genes can influence the lncRNA expression, subsequently affecting their function or structure, thereby impacting tumorigenesis and cancer prognosis [7–9].

HOTTIP is a lncRNA that is transcribed from the 5’ end of HOXA cluster, involved in the HOX gene network, and can influence cell function by targeting HOXA gene transcription, resulting in epigenetic alteration and promoting tumor occurrence and progression [10]. HOTTIP is considered an oncogene as its overexpression has been observed in different cancers, such as hepatocellular carcinoma (HCC) [11], pancreatic ductal adenocarcinoma [12], gastric cancer [13], and NPC [14].

Maternally expressed gene 3 (MEG3) is an imprinted gene, which encodes about 1.6 kb long non-coding RNA and has been indicated as a tumor suppressor. Much research has indicated that MEG3 expression is inhibited in different tumors, such as gastric [15], ovarian [16], and prostate cancers [17], as well as NPC [18]. Moreover, it has been revealed that HOTTIP up-regulation or MEG3 down-regulation is associated with cancer pathogenesis and progression.

Some studies have indicated the association of HOTTIP and MEG3 genetic polymorphisms with cancer susceptibility. For instance, Abdelaleem et al. revealed that HOTTIP rs1859168 variation was linked with an increased susceptibility to breast cancer (BC) and might be utilized as an indicator and therapeutic target for BC patients [19]. Wang et al. demonstrated that the rs3807598 and rs2067087 variants of HOTTIP were correlated with elevated gastric cancer risk [20]. Furthermore, Elhelaly revealed that MEG3 rs7158663 was linked with colorectal cancer (CRC) risk and therefore may serve as its diagnostic and prognostic index [21]. Mohammed et al. found that MEG3 rs7158663 promotes the risk of HCC and may be poor diagnostic and prognostic factors for HCC patients [22]. However, the associations of HOTTIP and MEG3 SNPs with NPC remain unclear. This investigation aimed to elucidate if rs1859168 and rs7158663 genetic variants are linked with increased NPC susceptibility or development and assess whether these polymorphisms could affect HOTTIP and MEG3 expressions and their possible correlations with the clinicopathological features.

## Materials and methods

### Enrolled participants

This research comprised 200 newly diagnosed NPC patients who were pathologically confirmed at the Department of Otolaryngology of Minzu Hospital of Guangxi Zhuang Autonomous Region (Guangxi, China) from January 2018 and December 2022. Additionally, 200 healthy individuals were also recruited as healthy controls from the physical examination center of the same hospital. Patients who underwent chemotherapy or radiotherapy before surgery or had other types of cancer were not selected in the NPC cohort. Furthermore, for the healthy cohort, individuals with cancer history or those who were related to the enrolled patients were excluded. Both the cohorts were sex, age, and residential area matched. Moreover, 48 NPC tumor tissues and 20 normal nasopharyngeal biospecimens were collected for RNA expression analysis. This investigation was authorized by the Ethics Committee of Minzu Hospital of Guangxi Zhuang Autonomous Region and signed consent was acquired from each participant before the study began.

### DNA isolation and genotyping

The acquired tissues and venous blood were kept at -80℃ before experiments. HOTTIP rs1859168 and MEG3 rs7158663 polymorphisms for both tissue and blood samples were identified *via* multiplex PCR and next-generation sequencing. Briefly, DNA was isolated from the samples *via* the TIANamp Genomic DNA Kit (Tiangen Biotech, Beijing, China), per the kit’s guide. The acquired DNA was then quantified, and purified and its integrity was assessed using Nanodrop one ultra-micro-spectrophotometer and agarose gel electrophoresis. Subsequently, PCR was carried out using primers synthesized by Qike Biotechnology (Beijing, China). Table 1 indicates the optimized reaction conditions. Sequencing and genotyping were conducted by Qike Biotechnology (Guangzhou, China) after electrophoretic confirmation of the sequencing products. The genotypes of HOTTIP rs1859168 were designated as A/A, A/C, or C/C, while MEG3 rs7158663 were designated as A/A, A/G, or G/G.

**Table 1:** Primer sequence and the reaction condition for HOTTIP rs1859168 and MEG3 rs7158663

SNP	Primer Sequence	PCR Reaction Conditions			Length
		Initial Step	Melt/Anneal/Extend	Elongate Step	
rs1859168	Forward:5’-GAGGAGCCACAAGCAGGTT-3’	94℃ for 3 min	35 cycles of 94℃ for 30 s, 57℃ for 30s, and 72℃ for 30 s	72℃ for 5 min	283 bp
	Reverse:5’-CCAAGACAAGAAGAGAACAAGC-3’				
rs7158663	Forward:5’-GGTGGGTTTGGGTTGGATGA-3’				263 bp
	Reverse: 5’-ACCAGACAGGAGCACTAGGAT − 3’				

### Quantitative real-time polymerase chain reaction (qRT-PCR)

Whole tissue RNA was acquired via the TRIzol reagent kit (Tiangen Biotech, Beijing, China), which was then quantified and purified before assessing its integrity using a Nanodrop one ultra-micro-spectrophotometer and agarose gel electrophoresis. Afterward, cDNA was prepared using the RevertAid cDNA Synthesis Kit (Thermo Fisher, Beijing, China), per the kit’s guide. Subsequently, an Applied Biosystems 7500 Real-Time PCR system was employed for qRT-PCR analysis. GAPDH was selected as endogenous control and the 2-^ΔΔCt^ algorithm was applied to quantify HOTTIP and MEG3 expressions.

The primers synthesized by Sangon Biotechnology (Shanghai, China) were as follows:

HOTTIP:

Forward: 5’-CCTAAAGCCACGCTTCTTTG-3’.

Reverse: 5’-TGCAGGCTGGAGATCCTACT-3’.

MEG3:

Forward: 5’-GGGAAGGGACCTCGAATGTG − 3’.

Reverse: 5’-CTGTCCCGTGGGAATAGGTG-3’.

GAPDH:

Forward: 5’-GTCAAGGCTGAGAACGGGAA-3’.

Reverse: 5’-AAATGAGCCCCAGCCTTCTC-3’.

### Statistical analysis

For statistical assessment, SPSS software v20.0 was utilized. The distribution frequencies of genotype and allele were compared and Hardy-Weinberg equilibrium (HWE) was elucidated by Chi-squared test. The relative risk related to various genotypes and alleles was elucidated by logistic regression analyses based on 95% confidence intervals (95% CIs) and calculated odds ratios (ORs). Furthermore, a non-parametric Mann-Whitney U test was carried out to compare the relative expressions of HOTTIP and MEG3 in tissue samples of different genotypic groups. A *p-value < 0.05* was deemed statistically significant.

## Results

### Clinical, demographic, and pathological features of the participants

Table 2 summarizes the comprehensive data on the NPC cases and control cohort. No marked differences in age, nationality, smoking, gender, and drinking proportion were identified between NPC patients and healthy individuals (*p > 0.05*). However, the EBV-DNA positive rate was substantially higher in NPC patients than in the controls (*p < 0.001*).

**Table 2:** Characteristics of the participants

Variables	Cases *n* (%)	Controls *n* (%)	*p*-value
Gender			
Male	151(75.30)	145(72.50)	0.494
Female	49(24.70)	55(27.50)	
Age, years			
Mean (SD)	51.09(9.52)	49.83(9.67)	0.378
≤ 50	84((42.00)	93(46.50)	0.365
> 50	116(58.00)	107(53.50)	
EBV-DNA			
positive	161(80.50)	9(4.50)	< 0.001
negative	39(19.50)	191(95.50)	
Nationality			
Han	108(54.00)	120(60.00)	0.226
Zhuang	92(46.00)	80(40.00)	
Smoking status			
Smoker	54(27.00)	41(20.50)	0.127
Nonsmoker	146(73.00)	159(79.50)	
Drinking status			
Drinker	79((39.50)	67(33.50)	0.213
Nondrinker	121(60.50)	133(66.50)	
Clinical stage			
I + II	102(51.00)	NA	NA
III + IV	98(49.00)	NA	
Local tumor invasion			
T1 + T2	128(64.00)	NA	NA
T3 + T4	72(36.00)	NA	
Lymph node involvement			
N0 + N1	134(67.00)	NA	NA
N2 + N3	66(33.00)	NA	
Distant metastasis			
Yes	23(11.50)	NA	NA
No	177(88.50)	NA	

Case, nasopharyngeal carcinoma patient; Control, healthy subject. *p*-value < 0.05 was set as the significant threshold

### Distribution frequency of the genotypes and alleles of rs7158663 and rs1859168 in NPC patients and healthy participants

**Fig. 1:**
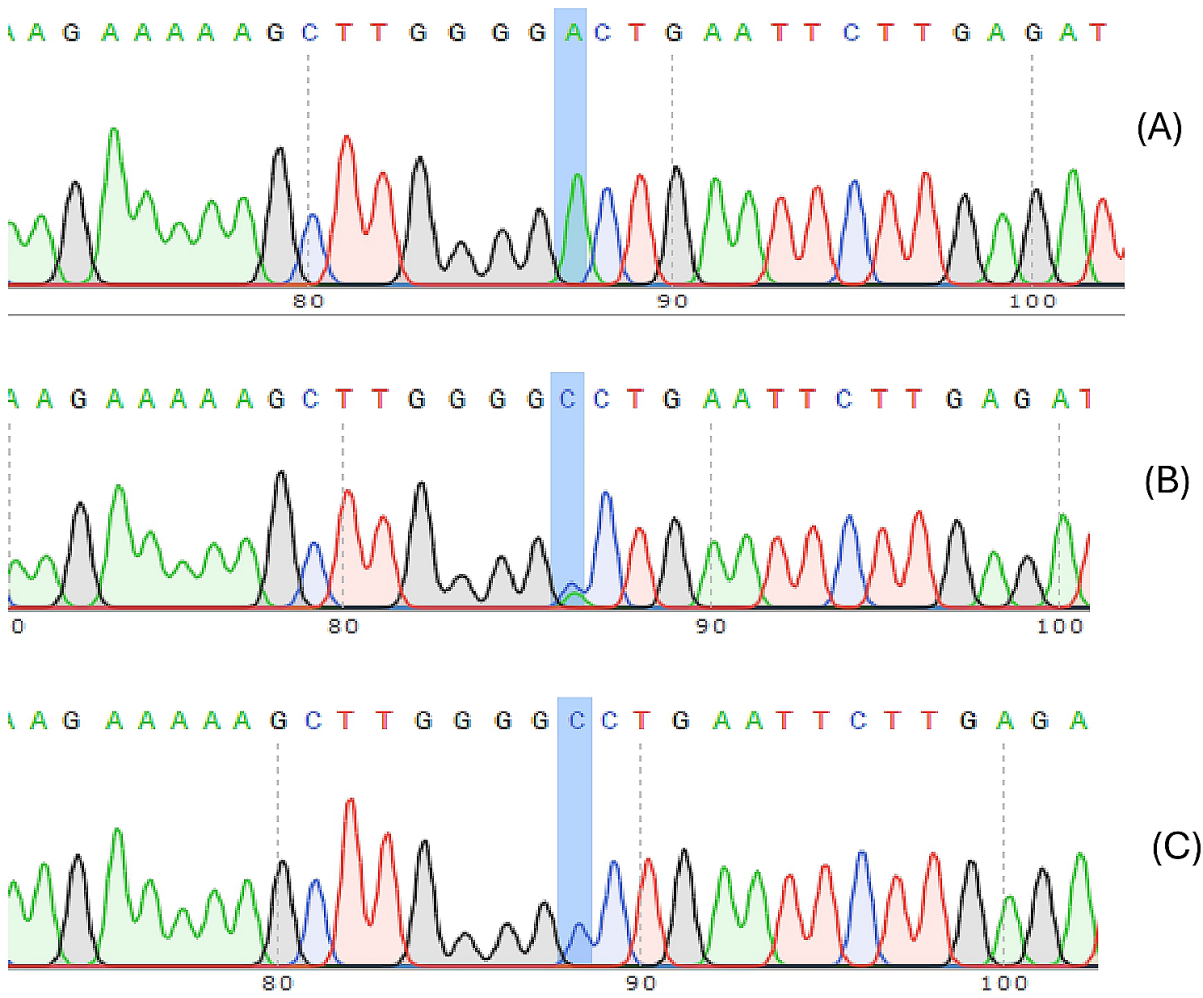
Genotype sequencing map of the HOTTIP rs1859168 polymorphism. (**A**) rs1859168- A/A; (**B**) rs1859168-A/C; (**C**) rs1859168-C/C

**Fig. 2:**
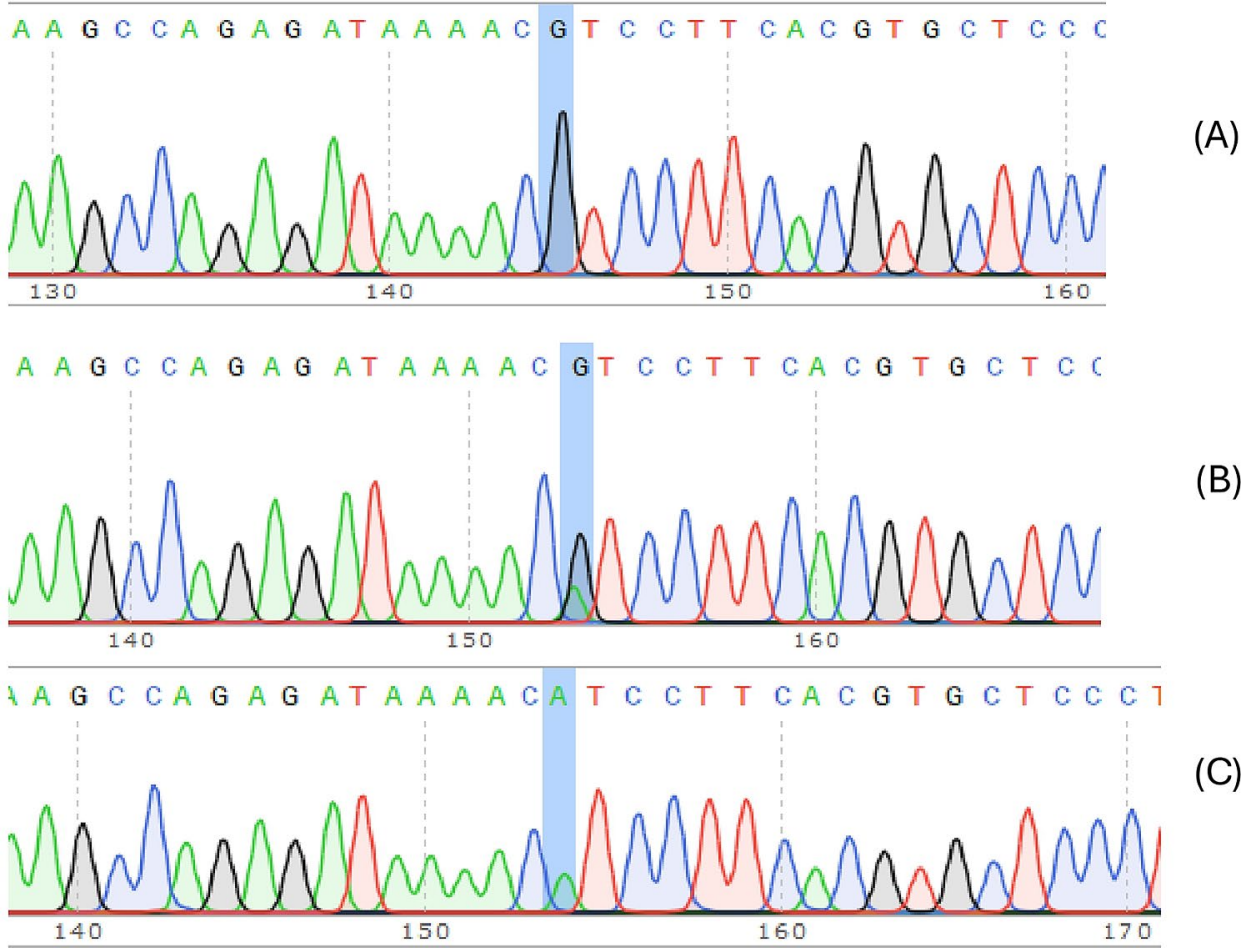
Genotype sequencing map of the MEG3 rs7158663 polymorphism. (**A**) rs7158663- G/G; (**B**) rs7158663-G/A; (**C**) rs7158663-A/A

Figures 1 and 2 present the genotyping sequencing data of rs1859168 and rs7158663. The distribution of these genotypes in patients and controls conformed to the Hardy-Weinberg equilibrium (all *p* > 0.05). Additionally, for the same cases, the genotypes of tissues were consistent with those of blood samples. The distribution of various alleles and genotypes of rs1859168 and rs7158663 are presented in Table 3.

The HOTTIP rs1859168 CC genotype was markedly linked with increased NPC incidence than the AA genotype (95% CI: 1.511–4.890, OR: 2.718, *p* = 0.001). Furthermore, the dominant model indicated a notable association of the AC + CC genotypes with increased NPC susceptibility (95% CI: 1.198–2.911, OR: 1.867, *p = 0.*005). Additionally, the recessive model revealed that the CC genotype was linked with a higher risk of NPC (95% CI: 1.201–3.284, OR: 1.991, *p* = 0.006). Moreover, the C allele was also related to an elevated risk of NPC (95% CI: 1.215–2.218, OR: 1.608, *p* = 0.001).

Compared with the MEG3 rs7158663 GG genotype, the AA genotype demonstrated substantial association with enhanced risk of NPC (95% CI: 1.194–4.007, OR: 2.187, *p* = 0.010). Furthermore, the GA + AA genotypes in the dominant model were notably linked with an enhanced NPC susceptibility (95% CI: 1.028–2.262, OR: 1.525, *p* = 0.036). Meanwhile, in the recessive model, the AA genotype showed a relation with increased NPC risk (95% CI: 1.088–3.442, OR: 1.935, *p* = 0.023). Moreover, the A allele was also linked with increased NPC risk (95% CI: 1.132–2.051, OR: 1.524, *p* = 0.005).

**Table 3:** Distribution frequencies of allele and genotype in patients and controls

SNPs		Cases *n* (%)	Controls *n* (%)	OR (95% CI)	*P*
HOTTIP rs1859168					
Genotype	AA	44(22.00)	69(34.50)	1.000	
	AC	104(52.00)	101(50.50)	1.615(1.012–2.575)	0.044
	CC	52(26.00)	30(15.00)	2.718(1.511–4.890)	0.001
Dominant Model	AA	44(22.00)	69((34.50)	1.000	
	AC + CC	156(78.00)	131(65.50)	1.867(1.198–2.911)	0.005
Recessive Model	AA + AC	148(74.00)	170(85.00)	1.000	
	CC	52((26.00)	30(15.00)	1.991(1.207,3.284)	0.006
Allele	A	192(48.00)	239(59.75)	1.000	
	C	208(52.00)	161(40.25)	1.608(1.215–2.128)	0.001
MEG3 rs7158663					
Genotype	GG	87(43.50)	108(54.00)	1.000	
	GA	76(38.00)	71(35.50)	1.329(0.865–2.041)	0.194
	AA	37(18.50)	21(10.50)	2.187(1.194–4.007)	0.010
Dominant Model	GG	87(43.50)	108(54.00)	1.000	
	GA + AA	113((56.50)	92(46.00)	1.525(1.028–2.262)	0.036
Recessive Model	GG + GA	163(81.50)	179(89.50)	1.000	
	AA	37(18.50)	21(10.50)	1.935(1.088,3.442)	0.023
Allele	G Allele	250(62.50)	287(71.75)	1.000	
	A Allele	150(37.50)	113(28.25)	1.524(1.132–2.051)	0.005

### Stratified analyses of genotype distributions by gender, age, EBV-DNA, nationality, status of smoking and drinking in NPC patients

Further stratified analysis indicated that gender, EBV infection, age, smoking, nationality, and drinking status do not affect the association of HOTTIP rs1859168 and MEG3 rs7158663 with NPC susceptibility (all *p* > 0.05) (Table 4). These datas suggest that potential confounding variables had few modification effects on NPC susceptibility related to rs1859168 and rs7158663 genotypes.

**Table 4:** Stratification analyses of rs1859168 and rs7158663 genotypes among NPC patients

Characteristics	HOTTIP rs1859168 Genotype *n* (%)				MEG3 rs7158663 Genotype *n* (%)			
	AA	AC + CC	*p*-value	OR (95% CI)	GG	GA + AA	*p*-value value	OR (95% CI)
Gender								
Male	34(22.52)	117(77.48)	0.757	1.133(0.513,2.504)	68(45.03)	83(54.97)	0.443	1.294(0.670,2.699)
Female	10(20.41)	39(79.59)			19(38.78)	30(61.22)		
Age, years								
≤ 50	16(19.05)	68(80.95)	0.391	1.352(0.678,2.699)	39(46.43)	45(53.57)	0.477	1.228(0.697,2.162)
> 50	28(24.14)	88(75.86)			48(41.38)	68(58.62)		
EBV-DNA								
Positive	32(19.88)	129(80.12)	0.141	1.792(0.819,3.918)	73(45.34)	88(54.66)	0.286	1.481(0.718,3.056)
Negative	12(30.77)	27(69.23)			14(35.90)	25(64.10)		
Nationality								
Han	25(23.15)	83(76.85)	0.671	1.157(0.590,2.271)	50(46.30)	58(53.70)	0.387	1.281(0.730,2.250)
Zhuang	19(20.65)	73(79.35)			37((40.22)	55(59.78)		
Smoking status								
Smoker	9(16.67)	45(83.33)	0.268	0.634(0.282,1.426)	21(38.89)	33(61.11)	0.424	0.771(0.408,1.458)
Nonsmoker	35(23.97)	111(76.03)			66(45.21)	80(54.79)		
Drinking status								
Drinker	14(17.72)	65(82.28)	0.238	0.653(0.321,1.329)	31(39.24)	48(60.76)	0.326	0.750(0.421,1.333)
Nondrinker	30(24.79)	91(75.21)			56(46.28)	65(53.72)		

### Associations of rs1859168 and rs7158663 genotypes with clinicopathological features of NPC patients

The association of different HOTTIP rs1859168 and MEG3 rs7158663 genotypes with the NPC clinicopathological features was assessed (Table 5).

It was revealed that in comparison with the HOTTIP rs1859168 AA genotype, patients carrying the CC genotype had a greater incidence of III + IV clinical stage (OR: 2.311, *p* = 0.044), T3 + T4 local tumor invasion (OR: 2.575, *p* = 0.027), N2 + N3 lymph node status (OR: 2.914, *p* = 0.017).

Furthermore, patients carrying MEG3 rs7158663 AA genotype had an increased risk of T3 + T4 local tumor invasion (OR: 2.771, *p* = 0.011) and N2 + N3 lymph node status (OR: 2.511, *p* = 0.023) than those with GG genotype.

**Table 5:** Correlation of rs7158663 and rs1859168 genotypes with clinicopathologic features of NPC

Genotype	Pathological parameters		*p*-value	OR (95% CI)
HOTTIP rs1859168	Clinical stage: I + II	Clinical stage: III + IV		
AA n (%)	26(59.09)	18(40.91)		1.000
AC n (%)	56(53.85)	48(46.15)	0.557	1.238(0.606,2.528)
CC n (%)	20(38.46)	32(61.54)	0.044	2.311(1.017,5.250)
	Local tumor invasion: T1 + T2	Local tumor invasion: T3 + T4		
AA n (%)	31(70.45)	13(29.55)		1.000
AC n (%)	72(69.23)	32(30.77)	0.882	1.060(0.491,2.289)
CC n (%)	25(48.08)	27(51.92)	0.027	2.575(1.105,6.000)
	Lymph node status: N0 + N1	Lymph node status: N2 + N3		
AA n (%)	34(77.27)	10(22.73)		1.000
AC n (%)	72(69.23)	32(30.77)	0.321	1.511(0.666,3.427)
CC n (%)	28(53.85)	24(46.15)	0.017	2.914(1.195,7.106)
	Metastasis: Yes	Metastasis: No		
AA n (%)	4(9.09)	40(90.91)		1.000
AC n (%)	9(8.65)	95(91.35)	0.932	0.947(0.276,3.255)
CC n (%)	10(19.23)	42(80.77)	0.161	2.381(0.691,8.209)
MEG3 rs7158663	Clinical stage: I + II	Clinical stage: III + IV		
GG n (%)	51(58.62)	36(41.38)		1.000
GA n (%)	36(47.37)	40(52.63)	0.151	1.574(0.847,2.927)
AA n (%)	15(40.54)	22(59.46)	0.065	2.078(0.950,4.545)
	Local tumor invasion: T1 + T2	Local tumor invasion: T3 + T4		
GG n (%)	63(72.41)	24(27.59)		1.000
GA n (%)	47(61.84)	29(38.16)	0.151	1.620(0.837,3.133)
AA n (%)	18(48.65)	19(51.35)	0.011	2.771(1.248,6.154)
	Lymph node status: N0 + N1	Lymph node status: N2 + N3		
GG n (%)	65(74.71)	22(25.29)		1.000
GA n (%)	49(64.47)	27(35.53)	0.155	1.628(0.830,3.196)
AA n (%)	20(54.05)	17(45.95)	0.023	2.511(1.120,5.630)
	Metastasis: Yes	Metastasis: No		
GG n (%)	8(9.20)	79(90.80)		1.000
GA n (%)	10(13.16)	66(86.84)	0.421	1.496(0.558,4.008)
AA n (%)	5(13.51)	32(86.49)	0.473	1.543(0.469,5.074)

### HOTTIP and MEG3 expression levels in NPC patients with different clinicopathological features

**Fig. 3:**
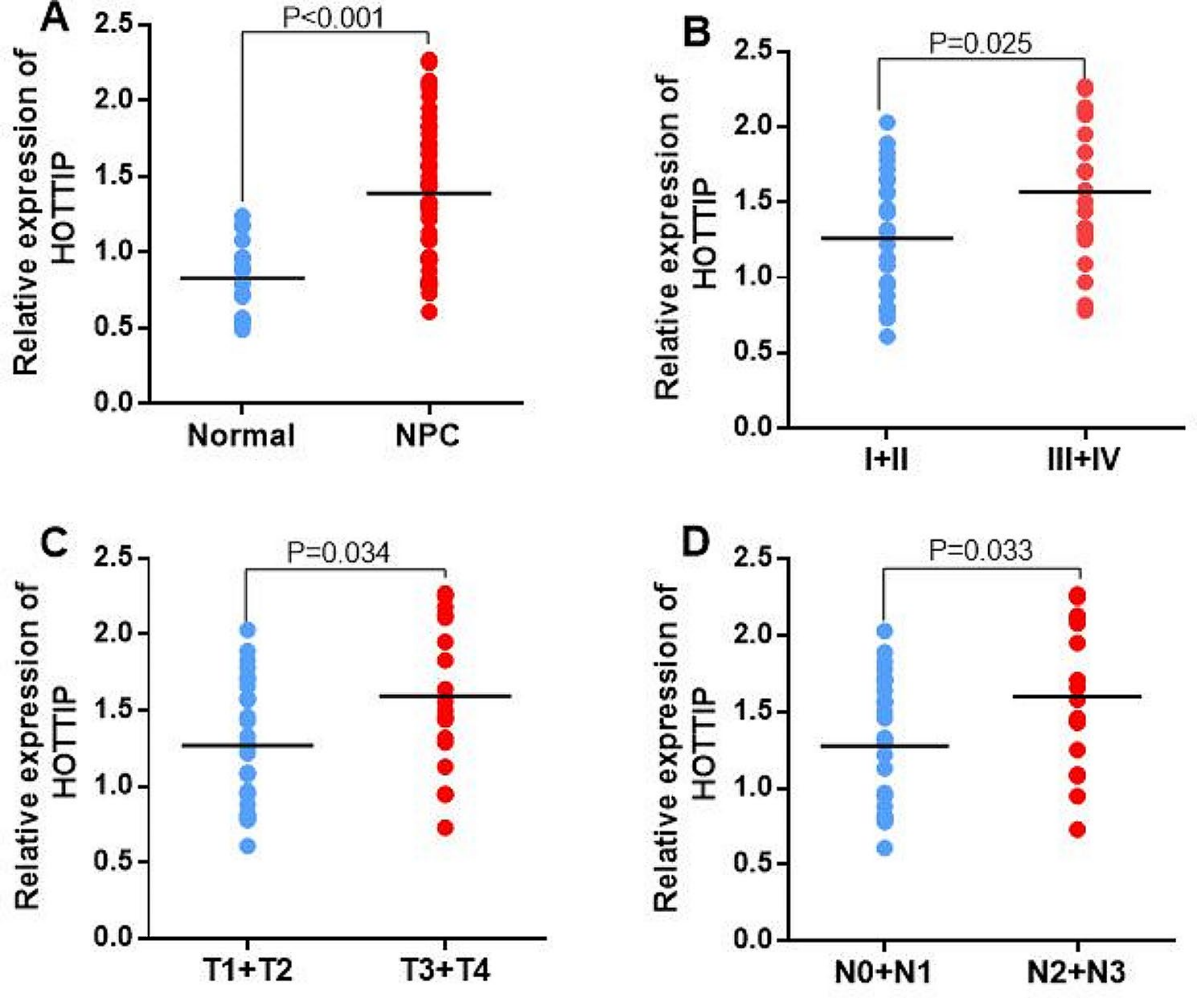
(**A**) The relative expression of HOTTIP in NPC tumor tissues and normal nasopharyngeal biospecimens. (**B**) The relative expression of HOTTIP in different clinical stages, (**C**) local tumor invasions, and (**D**) lymph node status

**Fig. 4:**
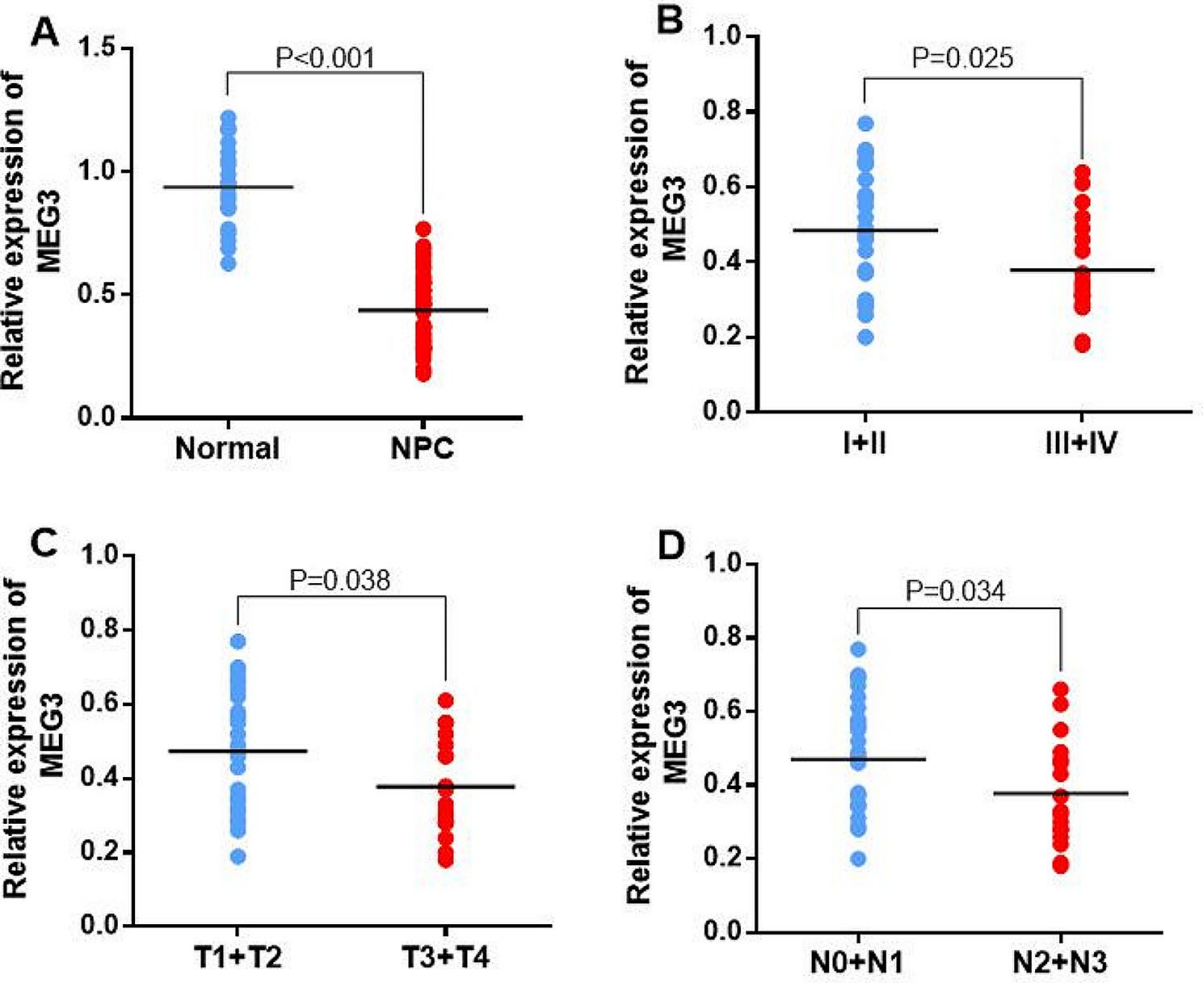
(**A**) The relative expression of MEG3 in NPC tumor tissues and normal nasopharyngeal biospecimens. (**B**) The relative expression of MEG3 in different clinical stages, (**C**) local tumor invasions, and (**D**) lymph node status

To understand how HOTTIP and MEG3 are related to NPC occurrence and progression, their expression levels in NPC tissues and their association with different clinicopathological parameters were assessed. It was revealed that HOTTIP had a higher expression in NPC tissues than control nasopharyngeal biospecimens (*p < 0.001*). Furthermore, the expression of HOTTIP was markedly elevated in NPC tissues with III + IV clinical stage, T3 + T4 local tumor invasion, and N2 + N3 lymph node status than those with I + II clinical stage, T1 + T2 local tumor invasion, and N0 + N1 lymph node status (all *p* < 0.05) (Fig. 3). Conversely, MEG3 indicated reduced expression in NPC patients than in healthy nasopharyngeal biospecimens (*p < 0.001*). Moreover, MEG3 indicated notably reduced expression in NPC tissues with III + IV clinical stage, T3 + T4 local tumor invasion, and N2 + N3 lymph node status than those with I + II clinical stage, T1 + T2 local tumor invasion, and N0 + N1 lymph node status (all *p* < 0.05) (Fig. 4).

### The effect of rs1859168 and rs7158663 on HOTTIP and MEG3 expressions in NPC

Subsequently, the association of different HOTTIP rs1859168 or MEG3 rs7158663 genotypes with HOTTIP or MEG3 expression in NPC patients was assessed.

**Fig. 5:**
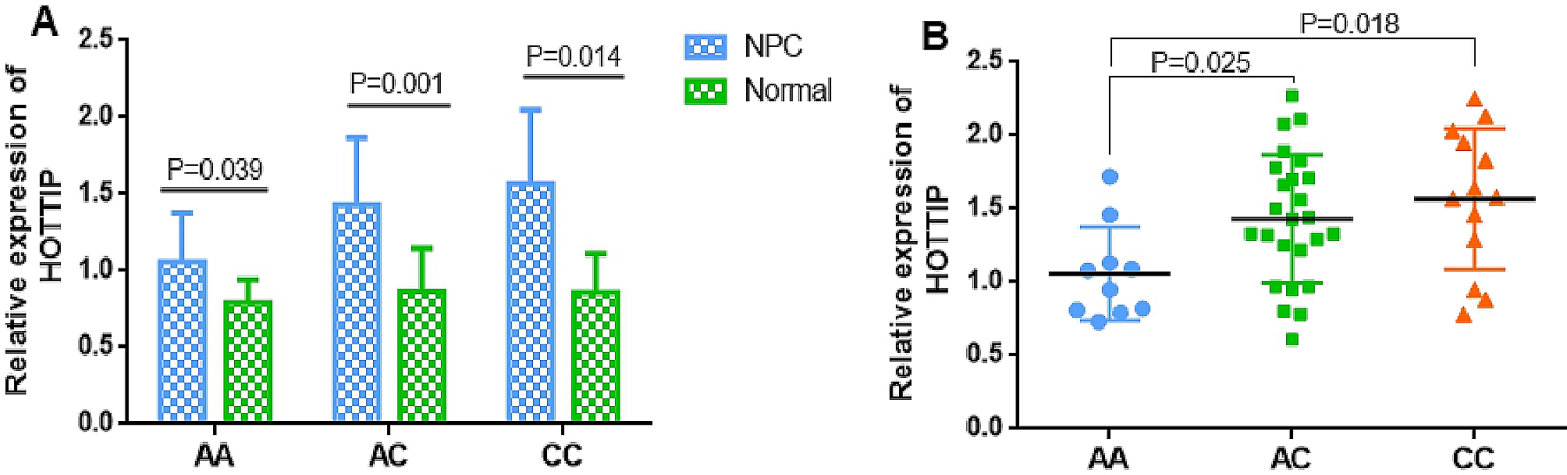
(**A**) The relative expression of HOTTIP of different genotypes in NPC tumor tissues and normal nasopharyngeal biospecimens. (**B**) The relative expression of HOTTIP of different genotypes in NPC tumor tissues

It was revealed that HOTTIP had a higher expression of different genotypes in NPC tissues than control nasopharyngeal biospecimens (Fig. 5A). Moreover, in comparison with the HOTTIP rs1859168 AA genotype, NPC patients carrying AC or CC genotypes exhibited notably higher levels of HOTTIP (Fig. 5B).

**Fig. 6:**
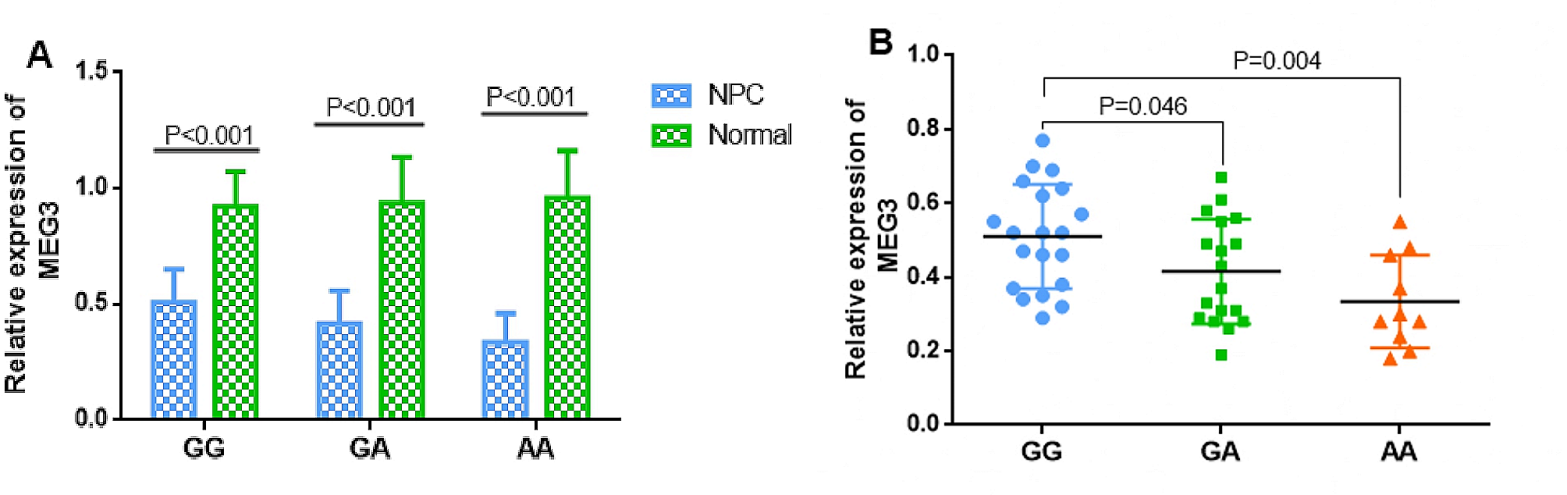
(**A**) The relative expression of MEG3 of different genotypes in NPC tumor tissues and normal nasopharyngeal biospecimens. (**B**) The relative expression of MEG3 of different genotypes in NPC tumor tissues

Furthermore, It was revealed that MEG3 had a lower expression of different genotypes in NPC tissues than control nasopharyngeal biospecimens (Fig. 6A). NPC patients carrying the rs7158663 GA or AA genotypes indicated markedly reduced levels of MEG3 than those with the GG genotype (Fig. 6B).

## Discussion

Nasopharyngeal carcinoma originates from the nasopharyngeal epithelium and is an aggressive type of neck and head cancer. Because of the narrow nasopharyngeal cavity, hidden onset, and deep tissue structure, at the time of diagnosis > 70% of patients have reached locoregionally advanced stages [23]. Therefore, identification of new genetic bio-indices and therapeutic targets is essential for early diagnosis and treatment. The research suggests that lncRNAs can serve as genetic bio-indices of NPC [24]. Furthermore, SNPs in lncRNA genes have been indicated to influence NPC susceptibility and progression [25, 26]. However, the association of HOTTIP rs1859168 and MEG3 rs7158663 polymorphism with NPC susceptibility and tumor progression remains undetermined. This current study is the first to indicate that the HOTTIP rs1859168 CC and MEG3 rs7158663 AA genotypes are markedly correlated with increased susceptibility to NPC and clinical pathological features. Moreover, these genotypes also influence HOTTIP and MEG3 expression in NPC patients.

HOTTIP is an antisense non-coding transcript located at the 5′end of the HOXA gene cluster and is considered an oncogene, valuable biomarker, and therapeutic target in various malignancies [11–13]. It also modulates the expression of cancer-associated genes at both transcriptional and post-transcriptional levels. For example, Lin et al. revealed that HOTTIP regulated HOXA13 gene transcription in the cells of esophageal squamous cell carcinoma, thereby influencing its tumorigenesis and metastasis [27]. Han et al. found that HOTTIP directly bonds to miR-148a-3p to increase WNT1 expression, facilitating BC stemness [28]. In addition, HOTTIP alters immune cell’s anti-cancer effects, thereby enhancing their immune evasion. For example, it has been observed to enhance IL-6 expression in ovarian cancer, consequently up-regulating PD-L1 expression in neutrophils, thereby accelerating tumor immune escape [29]. Two researches have elucidated the link between HOTTIP and NPC. Feng et al. reported that HOTTIP expression was markedly increased in NPC cells and tissues, and it promoted tumorigenesis by modulating the HOXA13 expression [30]. Shen et al. revealed that HOTTIP levels were markedly enhanced in NPC tissues and it promoted NPC cell’s ability to proliferate, migrate, and invade by sponging miR-4301 [14]. In the current study, it was revealed that HOTTIP expression was notably elevated in NPC tissues and it was positively related to local tumor invasion, clinical stage, and lymph node metastasis. Based on these results, HOTTIP might have a tumor-promoting role in NPC.

Many recent studies have indicated the associations of HOTTIP variants with cancer risk. Furthermore, these variants influenced HOTTIP expression by influencing the binding sites of transcription factors. According to Wang et al., HOTTIP rs2067087 and rs3807598 were related to increased risk of gastric cancer by affecting HOTTIP expression [20]. Another report revealed that the HOTAIR SNPs and their interactions with the HOTTIP rs1859168 polymorphism significantly increased the risk of gastric cancer [31]. Abdelaleem et al. suggested that individuals carrying the rs1859168 CC genotype had an elevated risk of BC as they influence HOTTIP and miR-615-3p levels [19]. Ali et al. demonstrated that rs1859168 C allele and CC genotype were associated with an elevated risk of CRC by up-regulating HOTTIP expression in the Egyptian population [32]. On the contrary, Duan et al. indicated that rs1859168 was markedly linked with an alleviated risk of gastric cancer in the Chinese population [33]. Moreover, according to Hu et al., the rs1859168 C allele and CC genotype notably reduced the susceptibility to pancreatic cancer by down-regulating the HOTTIP expression [34]. Thus, the tumor type and study population might be the primary factor influencing rs1859168 as a susceptibility marker for tumors. Here, the association of rs1859168 polymorphism with NPC risk was evaluated, which indicated that rs1859168 C allele and CC genotype notably increased NPC risk. Furthermore, the genotype-phenotype analysis showed that rs1859168 CC or AC genotype was related to the expression of HOTTIP, indicating that this variant might impact the HOTTIP expression by modulating its transcript function, thereby correlating with NPC risk.

It has been reported that MEG3 levels are reduced in many human malignancies, and it functions as a tumor suppressor [15–17], by modulating common cell signal transduction pathways. Yan et al. indicated that in retinoblastoma, MEG3 overexpression enhanced cell apoptosis and decreased cell proliferation and migration via PI3K/Akt/mTOR pathway inactivating [35]. Lv et al. exhibited that in non-small cell lung cancer, high MEG3 levels elevated miR-21-5p and PTEN expressions and inhibited cellular migration and invasion via the miR-21 5p/PTEN axis [36]. In most tumor types, MEG3 acts as a tumor suppressor by directly interacting with miRNAs or acting as their molecular sponge. For instance, Tao et al. revealed that MEG3 overexpression suppressed cell proliferation and invasion while stimulating apoptosis in ovarian cancer by sponging miR-205-5p [37]. According to Shan et al., MEG3 was notably reduced in bladder cancer cells, and it can alleviate the progression of bladder cancer by modulating PTEN and miR-494 [38]. Recently, Lin et al. demonstrated that in NPC cells, MEG3 levels were reduced, stimulating NPC cells’ autophagy and apoptosis by interacting with miR-21 to elevate PTEN expression [18]. Moreover, Zhou et al. proved that MEG3 was downregulated in NPC cells and tissues and its overexpression inhibited invasion, epithelial-mesenchymal transition, and migration of NPC cells by modulating SQSTM1 [39]. In accordance with the evidence mentioned previously, this current study was also found that MEG3 reduced notably in NPC tissues relative to normal nasopharyngeal biospecimens. In addition, MEG3 expression was negatively related to local tumor invasion, clinical stage, and lymph node metastasis in NPC.

Much evidence has indicated that MEG3 polymorphisms are linked with cancer susceptibility. rs7158663 is present on the MEG3 transcript and is the most interesting polymorphic locus. Bioinformatics assessments have indicated that rs7158663 polymorphism can alter the folding structure of local RNA and affect the interactions of miRNA-lncRNA, thereby affecting the levels of MEG3. Various research has elucidated the link between this polymerphism and cancer susceptibility. For example, Elhelaly et al. suggested that the rs7158663 AA genotype was related to CRC risk in Egyptian patients and might be utilized as a diagnostic and prognostic marker for CRC patients [21]. Gao et al. found that the rs7158663 GA or AA genotype was linked with an elevated risk of CRC in the Chinese population [40]. Per Kong et al., MEG3 rs7158663 AA or GA genotype and A allele carriers could substantially elevate gastric cancer risk [41], while according to Olfat et al., these carriers have increased susceptibility to BC [42]. However, some research indicated that the rs7158663 variant was not linked with cancer risk. Zhuo et al. revealed that the rs7158663 polymorphism was not related to neuroblastoma risk [43], while Yang et al. indicated that it was not linked with lung cancer susceptibility [44]. These inconsistent results may differ from tumor type and the study population. This is the first research to evaluate the association of MEG3 polymorphism with NPC, which revealed that the rs7158663 AA genotype was linked with an elevated risk of NPC, and the carriers of GA or AA genotypes had markedly reduced levels of MEG3. Therefore, it was speculated that the polymorphism of rs7158663 might affect an individual’s susceptibility to NPC by regulating MEG3 expression.

In addition, it has been indicated that SNPs in lncRNA genes influence the function of these lncRNAs and promote the progression of various tumors [45–47]. Few studies have provided evidence supporting the link between the HOTTIP rs1859168 and MEG3 rs7158663 genetic variants and the progression of tumors. Abdelaleem et al. revealed that in BC, the rs1859168 CC genotype and C allele were markedly linked with higher TNM staging [19]. Ali et al. also suggested that HOTTIP rs1859168 was substantially related to lymph node metastasis, distant metastasis, and grade III of CRC [32]. They also indicated that MEG3 rs7158663 polymorphism was notably linked with higher TNM staging and larger tumor size of BC in the Egyptian population [48]. Mohammed et al. verified that in HCC, the rs7158663 variant was markedly correlated with larger tumor size and advanced stage [22]. Here, the association of HOTTIP rs1859168 and MEG3 rs7158663 genetic variants with clinicopathological features of NPC patients was assessed. Patients carrying rs1859168 CC or rs7158663 AA genotypes had an increased risk of local tumor invasion and lymph node metastasis than rs1859168 AA or rs7158663 GG genotype carriers. These data suggests that HOTTIP rs1859168 and MEG3 rs7158663 variants might be associated with the NPC progression.

### Limitations

The limitations of this study include: (1) It is a case-control study, therefore is subjected to inevitable selection bias. (2) This investigation was executed as a single-center and requires further large-scale multicenter research to validate its data. (3) The molecular mechanisms of how rs1859168 and rs7158663 variants influence NPC pathogenesis and the link of these polymorphisms with HOTTIP and MEG3 expression in other ethnic groups need further elucidation.

## Conclusion

Altogether, it was revealed that HOTTIP rs1859168 and MEG3 rs7158663 genetic variants were related to an increased risk of NPC. Furthermore, rs1859168 CC or rs7158663 AA genotypes were associated with NPC patients’ clinicopathologic characteristics. Moreover, it was verified that the rs1859168 CC/AC or rs7158663 AA/GA genotypes were linked to high HOTTIP or low MEG3 levels in NPC patients. Comprehensive mechanisms of rs1859168 and rs7158663 variants influencing NPC pathogenesis warrant further research.

## Data Availability

The raw data supporting the results and conclusions of this article will be made available by the authors. All data generated or analyzed presented in the study are included in the article.
